# Phosphatase and tensin homolog (PTEN) expression on oncologic outcome in renal cell carcinoma: A systematic review and meta-analysis

**DOI:** 10.1371/journal.pone.0179437

**Published:** 2017-07-03

**Authors:** Lu Tang, Xintao Li, Yu Gao, Luyao Chen, Liangyou Gu, Jianwen Chen, Xiangjun Lyu, Yu Zhang, Xu Zhang

**Affiliations:** State Key Laboratory of Kidney Disease, Department of Urology, Chinese PLA Medical Academy, Chinese People’s Liberation Army General Hospital, Beijing, China; Seconda Universita degli Studi di Napoli, ITALY

## Abstract

The phosphatase and tensin homolog (*PTEN*) gene is suggested to be a dormant tumor suppressor. However, the prognostic value of the loss of *PTEN* expression in renal cell carcinoma (RCC) remains controversial. Therefore, we conducted a meta-analysis to evaluate the association of *PTEN* expression with the clinicopathological presentations and outcomes of patients with RCC through immunohistochemistry staining analysis. We systematically searched for relevant studies in PubMed, Web of Science, and Embase until March 2016. Data regarding clinical stage, pathological type, Fuhrman grade, overall survival (OS), progression-free survival (PFS), and disease-specific survival (DSS) was analyzed in the present study. In total, there were 12 studies with 2,368 patients included in this meta-analysis. The low *PTEN* expression in RCC was significantly associated with unfavorable DSS (HR = 1.568, 95% CI 1.015–2.242) in a random-effects model but not with OS (HR = 1.046, 95% CI 0.93–1.176) and PFS (HR = 1.244, 95% CI 0.907–1.704). Other results indicated that *PTEN* expression was not correlated with clinical stage, pathological type, and Fuhrman grade. This meta-analysis suggests that *PTEN* expression is of limited value in predicting the prognosis of patients with RCC for OS and PFS via immunohistochemistry staining analysis; and that for DSS, low *PTEN* expression is significantly associated with an unfavorable outcome.

## Introduction

Renal cell carcinoma (RCC) is one of the most common malignancies in urological neoplasms, accounting for 2%–3% of all adult malignancies [1]. Approximately 25%–30% of metastatic lesions are detected at initial diagnosis, and metastatic RCC (mRCC) is resistant to treatment [2, 3]. Although significant advancements in the understanding of RCC and improvements in its therapeutic strategies have been achieved in the past several decades, the vast majority of patients with advanced RCC still die of their disease rather than competing causes.

The discovery and validation of biomarkers that can indicate clinical behavior and provide information about the prognosis of a given tumor are crucial to successful oncotherapy. In particular, biomarkers that can distinguish an aggressive phenotype may guide the selection of intensive treatment in clinical practice [4]. It is thought that biomarkers have the potential to inform risk stratification, estimate treatment effects, and predict prognosis.

The phosphatase and tensin homolog is a protein encoded by the *PTEN* gene. *PTEN* maps to chromosome 10q23.3 and has been thoroughly investigated as a dormant tumor suppressor [5–7]. PTEN has been demonstrated to be a component of signal transduction pathways involved in the regulation of cell growth, proliferation, and apoptosis, as well as a participant in the control of the cell cycle [8–10]. For example, PTEN was shown to antagonize the phosphoinositol-3-kinase (PI3K)/PTEN/AKT signaling pathway, which plays a crucial role in cell growth, differentiation, and survival, thereby safeguarding important cellular machineries against carcinogenesis. Thus, the loss of *PTEN* expression may cause the initiation of tumorigenesis [11–15].

Consequently, investigating the association between *PTEN* expression and the survival outcomes of patients with RCC is important and helpful in adopting personalized treatment measures. The loss of the *PTEN* gene, which is a master cellular regulator, is associated with tumor progression and adverse outcomes in various human cancers [16–19]. Although *PTEN* has been thoroughly investigated, the prognostic value of the loss of its expression in RCC remains controversial because previous reports were inconsistent [20–23]. Some studies suggested that the loss of *PTEN* expression demonstrated an adverse association with prognosis, but other evidence showed no correlation between the two factors [15, 23, 24]. Thus, we performed a meta-analysis to evaluate the practicability of *PTEN* expression as a biomarker in RCC.

## Materials and methods

### Search strategy selection criteria

We systematically searched for relevant studies in PubMed, Web of Science, and Embase until March 2016. “PTEN,” “phosphatase and tensin homolog,” “renal or kidney,” “neoplasm, tumor, cancer, or carcinoma,” and “prognosis, outcome, progress, mortality, and survival” were used as search terms. The start date of our searches was 2003 and the end date was 2015. The reference lists of relevant articles were examined for additional eligible studies.

The titles and abstracts of relevant studies were scrutinized to exclude irrelevant articles. The relevant articles were reviewed as full texts afterward. Studies were included in this meta-analysis if they met the following inclusion criteria: (1) investigated the clinical presentations and prognosis of patients with RCC; (2) detected PTEN protein expression intensity in immunohistochemistry staining analysis; and (3) evaluated the correlation between PTEN protein expression and clinical presentations as well as survival outcomes [overall survival (OS), disease-specific survival (DSS) and (disease-free survival (DFS)]. The exclusion criteria were: (1) papers not written in English; (2) case reports, review articles, conference abstracts, or editorial comments; and (3) determination of *PTEN* expression by other methods, including RT-PCR and Western blot analysis.

The selection process was performed by two reviewers independently to ensure that the studies included were eligible. When studies contained duplicated data, the study with the largest sample size was chosen. Multivariate analysis outcomes were given preference over univariate results; if no multivariate results were provided, univariate outcomes were accepted, and the survival curves in included studies were used for calculation.

### Data extraction

Two investigators extracted data from included studies independently. The Newcastle–Ottawa Quality Assessment Scale (NOS) was used to assess the quality of each included study. Selection of study population, data comparability, and outcome in these cases and control groups were involved in this assessment scale. Studies with an NOS score ≥6 were defined as high-quality [25]. The following information was extracted from the included studies: the name of the first author, publication year, patient number, country, follow-up duration, antibody used, cutoff score, tumor pathological type, tumor clinical stage, Fuhrman grade, patient type (localized or metastatic RCC), DFS, DSS, OS, and *PTEN* expression level. In addition, we used GetData Graph Digitizer 2.26 (http://getdata-graph-digitizer.com/) to digitize and extract the survival data from a Kaplan–Meier curve given in some studies.

The cutoff value to define *PTEN*-low or *PTEN*-high expression varied across the included studies. The criteria described in the original articles were adopted.

### Statistical analysis

All statistical analyses were conducted using Stata version 12.0 (StataCorp LP, TX) software. Pooled ORs (odds ratio) with 95% CI (confidence interval) were calculated to measure the correlation between *PTEN* expression level and clinical stage and Fuhrman grade of RCC. Pooled RRs (risk ratio) with 95% CI were adopted to evaluate the correlation between *PTEN* expression and survival outcomes including DFS, DSS, and OS. The Q test and I^2^ test were used to measure heterogeneity among studies. A fixed-effects model or a random-effects model was used depending on the heterogeneity. If the heterogeneity was significant, a random-effects model was used; otherwise, a fixed-effects model was adopted. Publication bias was evaluated by Begg’s and Egger’s tests. All P values were two-tailed, and P < 0.05 was considered statistically significant. Data concerning geographic area, staining pattern, staining cutoff, tumor pathological type, patient type (localized or metastatic), sample size, and follow-up time was used in subgroup analyses to investigate the correlation between *PTEN* expression level and survival outcomes. The prognostic value of *PTEN* expression level in terms of clinical stage, Fuhrman grade, and pathological type was measured in only two, three, and three studies, respectively; thus, pooled analyses were conducted on these factors. A sensitivity analysis was performed using the leave-one-out approach to measure the reliability of the pooled results. This systematic review and meta-analysis adhered to the Preferred Reporting Items for Systematic Reviews and Meta-Analyses (PRISMA) guideline (Table A in S1 File).

## Results

### Literature search

**Fig 1:**
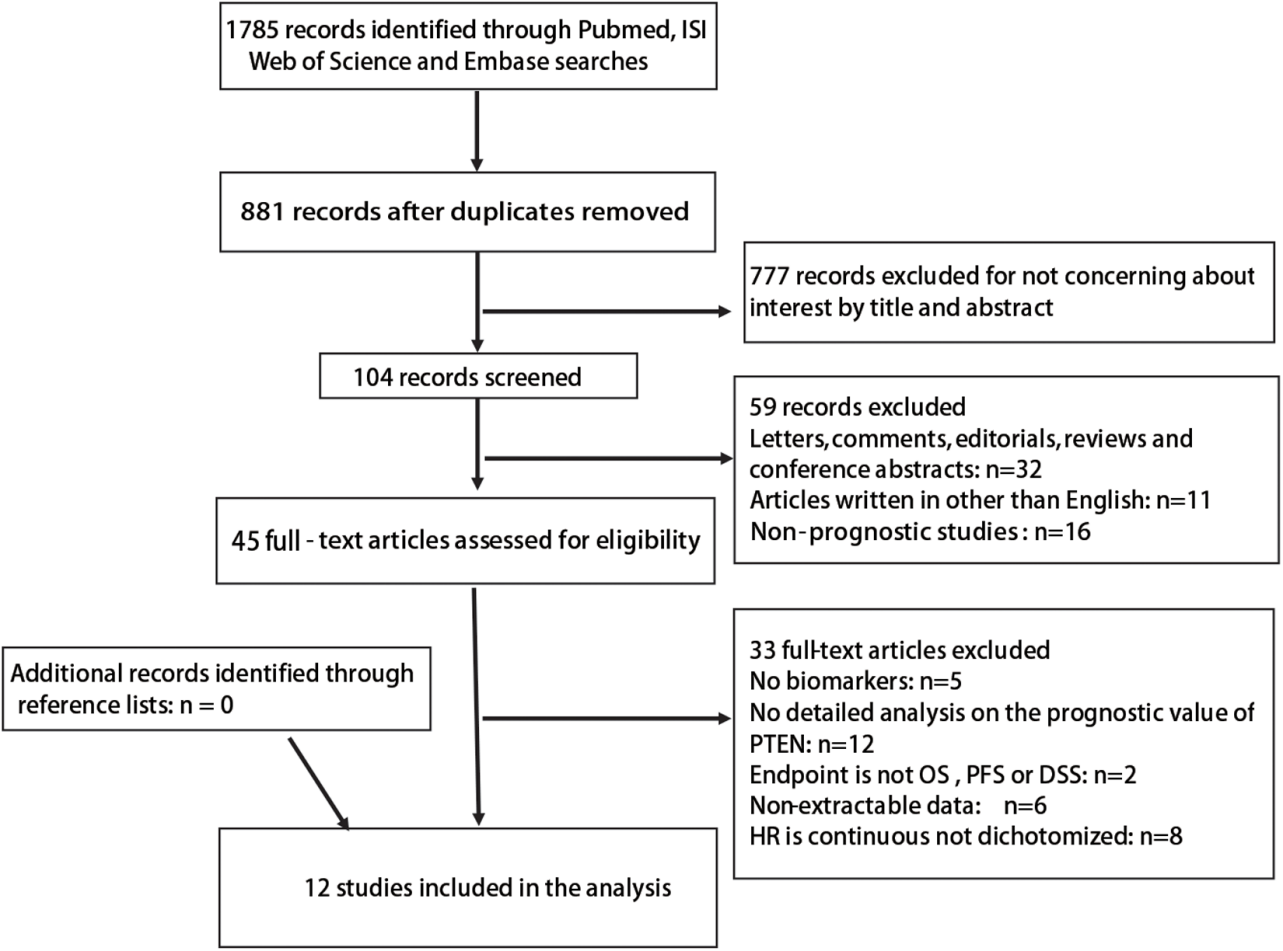
Flowchart of selection literature.

A flow chart of study selection is shown in Fig 1. A total of 1,785 records were obtained with the described search strategy. A total of 881 records were screened after exclusion of duplicates. Among these, 777 were excluded by reviewing the title and abstract. Of the remaining 104 records, 59 were excluded, including 32 letters, comments, reviews, and conference abstracts, 11 non-English articles, and 16 non-prognostic studies. The 45 remaining articles were assessed in full text. Of those, 33 articles were excluded, including: five that lacked of biomarkers; 12 missing detailed analysis on the prognostic value of PTEN; four whose endpoint was neither OS, PFS, nor DSS; six that had no extractable data; and eight in which HR was not dichotomized. A total of 12 studies with 2,368 patients were included in this meta-analysis. All the included studies investigated the association of *PTEN* expression with the clinicopathological presentations and outcomes of patients with RCC through immunohistochemistry staining analysis.

### Characteristics of eligible studies

The baseline characteristics of the 12 studies are summarized in the supporting information (Table B in S1 File). Among the included studies, four were performed in America, two in Japan, two in Korea, one in China and one in Austria. The median sample size was 130 patients with RCC (range: 33–528). The median follow-up duration was 55.75 months (range: 13.8–63.5). The included patients all underwent radical or partial nephrectomy. *PTEN* expression was detected in immunohistochemistry staining analysis in the included studies. The survival outcome of these patients was reported in 12 articles: among them, OS was reported in five studies and PFS and DSS were presented in five articles. The clinical stage was investigated in two studies, while five studies reported the type of selected patients (localized or metastatic). Five studies investigated *PTEN* expression among pathological types in RCC, and three reported the Fuhrman grading. The lymph node metastasis and TNM stage were both investigated in one study. Para-carcinoma, benign renal tumors, and normal renal specimens were also evaluated by comparing three studies discussing RCC.

### Effects of *PTEN* expression level on survival outcomes

**Fig 2:**
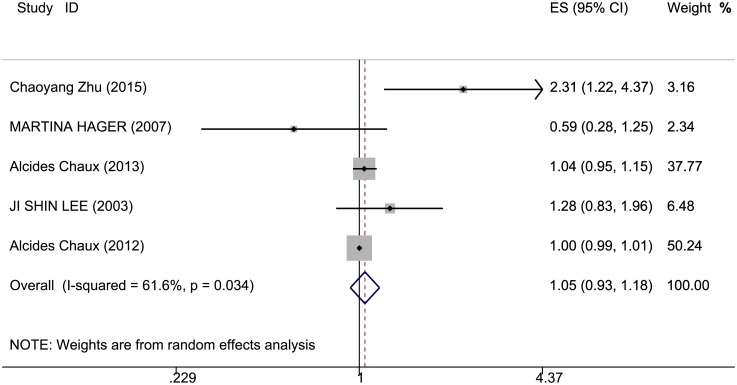
Meta-analysis results on *PTEN* expression and OS. Forest plot of the association between *PTEN* expression and overall survival of patients with RCC.

**Fig 3:**
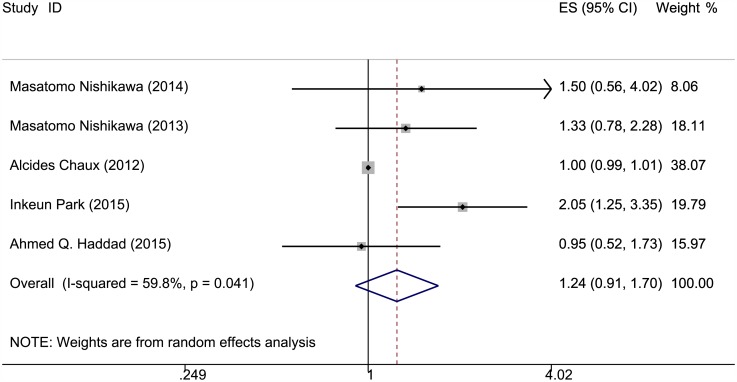
Meta-analysis results on *PTEN* expression and PFS. Forest plot of the association between PTEN expression and progression-free survival of patients with RCC.

**Fig 4:**
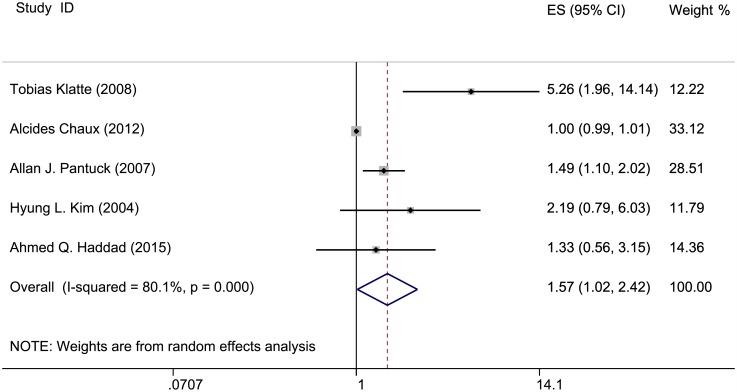
Meta-analysis results on *PTEN* expression and DSS. Forest plot of the association between PTEN expression and disease-specific survival of patients with RCC.

The correlation of *PTEN* expression and survival outcomes of patients with RCC is shown in Figs 2–4 and Table 1. OS, PFS, and DSS data were extracted from five studies. The *PTEN* expression level was not statistically significantly when associated with OS and PFS in the random-effects model (RR = 1.046, 95% CI = 0.93–1.176; RR = 1.244, 95% CI = 0.907–1.704); however, significant heterogeneity was observed among these studies (I^2^ = 61.6%, P = 0.034; I^2^ = 59.8%, P = 0.041). A lower *PTEN* expression was observed with significantly poorer DSS (RR = 1.568, 95% CI = 1.015–2.422); heterogeneity was significant in these studies as well (I^2^ = 80.1%, P = 0) (Table C in S1 File).

**Table 1:** Results of meta-analysis on *PTEN* expression.

	Overall survival (Survival vs. Death)				Progression free survival (Survival vs. Death)				Disease specific survival (Survival vs. Death)			
	n	HR	LCI	UCI	n	HR	LCI	UCI	n	HR	LCI	UCI
**Overall**	5	1.046	0.93	1.176	5	1.244	0.907	1.704	5	1.568	1.015	2.422
**Geographic area**												
1. Asian	2	1.637	0.925	2.897	3	1.66	1.181	2.332	NA	NA	NA	NA
2. non-Asian	3	1.005	0.96	1.053	2	1	0.99	1.01	NA	NA	NA	NA
**Staining pattern**												
1. cytoplasm	4	1.056	0.944	1.181	NA	NA	NA	NA	NA	NA	NA	NA
2.cytoplasm,nuclear	1	0.59	0.279	1.247	NA	NA	NA	NA	NA	NA	NA	NA
**Cutoff of staining**												
1. <50%	3	1.237	0.637	2.401	4	1.433	1.014	2.025	1	1	0.561	3.154
2. >=50%	2	1	0.991	1.01	1	1	0.99	1.01	4	1.64	1	2.691
**Sample size**												
1. <100	4	1.056	0.944	1.181	2	1	0.99	1.01	1	1	0.99	1.01
2. >=100	1	0.59	0.279	1.247	3	1.41	0.911	2.181	4	1.962	1.15	3.347
**Follow-up**												
1. <60	3	1.314	0.89	1.94	2	1.075	0.644	1.796	4	1.962	1.15	3.347
2. >=60	1	1	0.99	1.01	3	1.331	0.843	2.102	1	1	0.99	1.01
**Patient type**												
1. localized	NA	NA	NA	NA	1	1.33	0.778	2.274	NA	NA	NA	NA
2. metastatic	NA	NA	NA	NA	2	1.926	1.241	2.989	NA	NA	NA	NA
**Pathological type**												
1. clear cell RCC	NA	NA	NA	NA	1	0.95	0.521	1.733	2	1.639	0.849	3.166
2. other type RCC	NA	NA	NA	NA	1	1	0.99	1.01	2	2.124	0.42	10.735

HR, hazard ratio; LCI, lower confidence interval; UCI, upper confidence interval.

In terms of PFS, the subgroup meta-analysis showed that the cutoff scores under 50% indicated that lower *PTEN* expression was associated with poorer PFS (RR = 1.43, 95% CI = 1.014–2.025, I^2^ = 23.3) and that lower *PTEN* expression was observed with poor PFS in Asian areas of the geographic area subgroup (RR = 1.66, 95% CI = 1.181–2.332, I^2^ = 0). Lower *PTEN* expression was also observed with poor PFS in patients with metastatic RCC (RR = 1.926, 95% CI = 1.241–2.989, I^2^ = NA). For DSS, in over 50% of the cutoff scores of the subgroup, lower *PTEN* expression was observed with poorer DSS (RR = 1.64, 95% CI = 1–2.691, I^2^ = 84.8), and a sample size of ≥100 showed a significant difference among the *PTEN* expression levels (RR = 1.962, 95% CI = 1.15–3.347, I^2^ = 52.1). The follow-up subgroup under 60 months showed poorer DSS with lower *PTEN* expression (RR = 1.962, 95% CI = 1.15–3.347, I^2^ = 52.1).

### Correlations between *PTEN* expression and clinicopathological presentations

The associations between *PTEN* expression level and the clinical presentations of RCC are listed in Table 2. Respectively, three, two, and three studies were available for pooled analyses in terms of clinical stage, pathological type, and Fuhrman grade. However, no significant correlations were observed between *PTEN* expression levels and clinical stage, pathological type (ccRCC vs. other type), or Fuhrman grade in our meta-analysis (OR = 1.918, 95% CI = 0.502–7.336; OR = 0.781, 95% CI = 0.249–2.453; OR = 2.415, 95% CI = 0.508–9.055).

**Table 2:** Meta-analysis results of *PTEN* expression level and clinical features.

	n	OR	LCI	UCI	Heterogeneity		Publication bias	
					Pa	I^2^ (%)	Pc (Begg's test)	Pd (Egger' test)
**Clinical stage** Stage I+II vs. III+IV	2	1.918	0.502	7.336	0.021	81.3	0.317	NA
**Pathological type** ccRCC vs. Other type	3	0.781	0.249	2.453	0.017	75.3	0.602	0.396
**Fuhrman grade** I+II vs. III+IV	3	2.145	0.508	9.055	0	87.6	0.602	0.084

OR, odds ratio; LCI, lower confidence interval; UCI, upper confidence interval; ccRCC, clear cell renal cell carcinoma.

### Publication bias analysis

**Fig 5:**
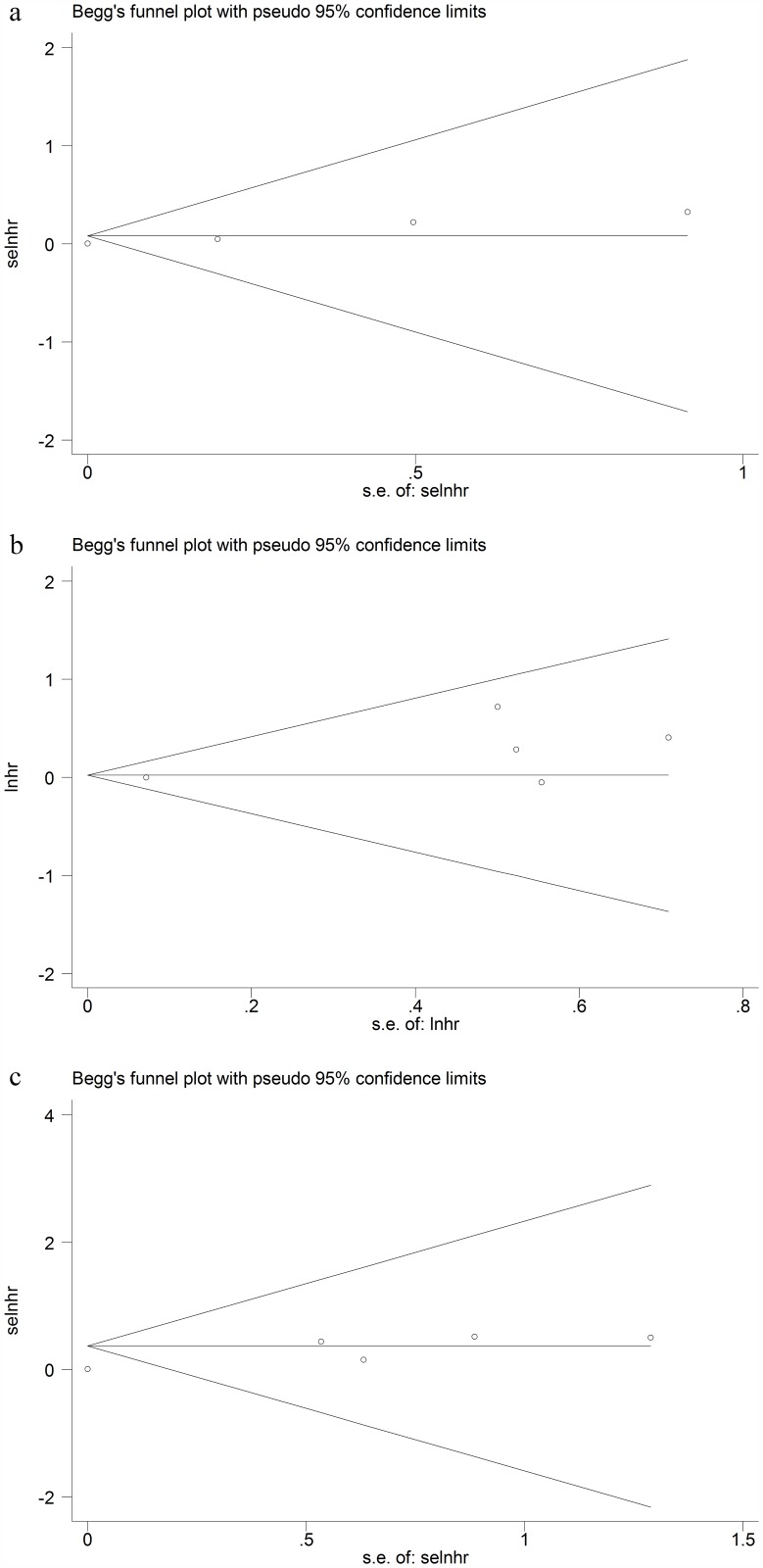
Begg's funnel plots of publication bias for OS, PFS and DSS. a. Begg's funnel plot for OS; b. Begg's funnel plot for PFS; c. Begg's funnel plot for DSS.

Begg’s and Egger’s tests were adopted to detect potential publication bias for OS, PFS, and DSS. No significant publication bias was observed in funnel plot asymmetry on OS, PFS and DSS. (Begg’s P value = 0.624, Egger’s P value = 0.557; Begg’s P value = 0.624, Egger’s P value = 0.158; Begg’s P value = 0.142, Egger’s P value = 0.057) (Fig 5). The results demonstrated that the included studies were almost symmetric.

### Sensitivity analysis

**Fig 6:**
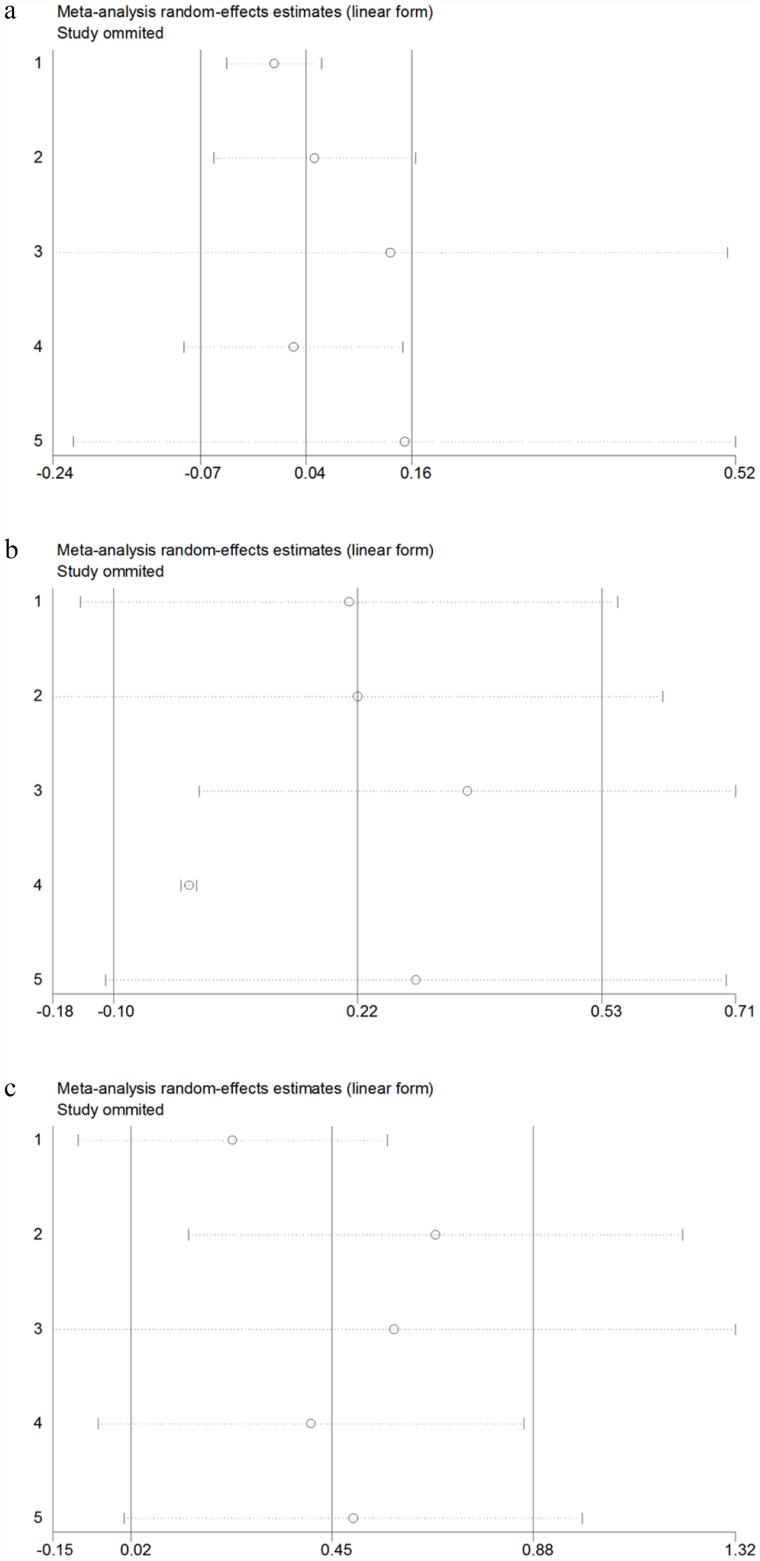
Sensitivity analysis for OS, PFS and DSS. a. Sensitivity analysis for OS; b. Sensitivity analysis for PFS; c. Sensitivity analysis for DSS.

A sensitivity analysis was conducted using the leave-one-out approach to measure the robustness of the pooled HRs for OS, PFS, and DSS (Fig 6). The results did not differ significantly from primary results, suggesting that the pooled results were reliable.

## Discussion

The present meta-analysis focused on the association between *PTEN* expression in immunohistochemistry staining analysis and the outcomes of patients with RCC. Overall, *PTEN* expression provides a significant prognostic value in DSS: patients with lower *PTEN* expression showed shorter DSS. No significant predictive value was detected for OS and PFS. Thus, the low *PTEN* expression in immunohistochemistry staining analysis presented limited value in predicting the OS and PFS. The vast majority of patients, particularly those with advanced RCC, still die of their disease rather than competing causes. Thus, *PTEN* expression may be used as a marker for postoperative prognosis to predict the disease specific survival. The results of subgroup analysis for OS were consistent. In terms of PFS, patients with lower *PTEN* expression presented significantly worse outcomes in the patients with metastatic RCC. However, for patients with localized RCC, no significant association was observed between the *PTEN* expression level and patient survival. No publication bias was detected. Positive *PTEN* was indicative of a good prognosis for receiving vascular endothelial growth factor receptor tyrosine kinase inhibitors (VEGFR TKIs) in patients with advanced RCC [26–28]. These results further demonstrated that the *PTEN* expression level can be a useful biomarker for treatment planning in advanced RCC.

When the cutoff of staining was below 50%, lower *PTEN* expression exhibited a worse outcome for PFS, whereas when the cutoff staining was over 50%, the association was absent. This result demonstrated that RCC with low *PTEN* expression was likely to exhibit worse prognosis. In terms of intracellular localization, *PTEN* expression was present predominantly in the cytoplasm of RCC cells, while only a small fraction was present in the nuclear and membrane; as such, the *PTEN* expression pattern of RCC is not prognostic for patient survival [24]. Brenner, *et al*. reported that membrane PTEN is lost in early-stage renal cell carcinogenesis and may be used as a valuable tumor marker [20]. In sample size and follow-up duration subgroup analysis, *PTEN* expression was correlated with the observed result of DSS. When the sample size was ≥100, the low *PTEN* expression presented a significant prognostic value for DSS. However, the small sample size group was not observed to exhibit worse DSS at low expression levels. The absence of significance may be attributed to the limited sample size.

Our findings indicate that *PTEN* expression was not correlated with clinical stage (stage I+ II vs. stage III+IV), pathological type (clear cell RCC vs. other RCC types), and Fuhrman grade (I+II vs. III+IV). This result demonstrated that the PTEN expression level is not correlated with these factors, which indicated a prognostic significance [29, 30]. Thus, the *PTEN* expression may not reflect the risk stratification in the clinical practice of RCC. Some studies reported that the lowest total and cytoplasmic *PTEN* expression levels were detected in clear-cell carcinoma and not in other subtypes [28, 31]. Some studies also showed that the *PTEN* expression was not associated with lymph node metastasis, tumor size, or patients’ age and gender [23, 32–34]. These results were consistent with those obtained in the current analysis.

*PTEN* is one of the most frequently mutated genes in human tumors, and its mutational status was identified in various cancers [35, 36]. For instance, approximately 40% of prostate cancer patients exhibited *PTEN* mutations, and the frequency for patients with castration-resistant prostate cancer (CRPC) was shown to be higher. PTEN loss was associated with shorter progression-free survival time [37]. In the observations of gliomas, *PTEN* mutations were restricted to high-grade gliomas. However, no obvious relationship was observed between the prognosis outcome and *PTEN* mutation status [38]. High *PTEN* mutation frequency was also observed in patients with breast cancer and gastric cancer [39, 40]. *PTEN* mutations occur in ccRCC, where they have been demonstrated to be loss-of-function mutations occurring in 1% to 5% of cases [41]. Evidence showed that the lifetime risks for RCC were projected to be approximately 30% with germline *PTEN* mutation [35]. Lee, *et al*. reported that *PTEN* biallelic loss was associated with an adverse ccRCC outcome, whereas monoallelic loss was not associated with poor prognosis [42]. Velickovic, *et al*. also reported poor prognosis in RCC patients with *PTEN* loss of heterozygosity [43]. de Campos, *et al*. reported that the deletion of *PTEN* in RCC was detected with a frequency of approximately 40% via fluorescent in situ hybridization and that its presence did not indicate lower survival rates [44]. Thus, *PTEN* mutations and loss of heterozygosity were demonstrated to play a key role in the etiology of various cancers and were associated with late-stage diseases [45]. The expression of *PTEN* was demonstrated to be evidently lower than that of the corresponding normal renal tissues in ccRCCs by Western blot analysis [20]. Zhu, *et al*. observed that *PTEN* expression decreased in normal renal tissues, para-carcinoma tissues, and malignant tissues [23]. These results may indicate that the loss of PTEN is involved in the entire carcinogenesis of RCC. Schneider, *et al*. suggested that an increased level of *PTEN* expression indicated a lower risk for developing metastasis, which corresponded to our results [46]. Merseburger, *et al*. detected no correlation between *PTEN* expression and patient survival [47]. Our findings showed that low *PTEN* expression levels corresponded to significantly shorter DSS, whereas in OS and PFS, the tendency did not reach statistical significance. Thus, PTEN as a biomarker seems to have limited usefulness in prognosis prediction.

Several limitations in our analysis should be considered. First, the retrospective studies were more susceptible to selection bias. Second, the cutoff value to define *PTEN*-high or *PTEN*-low expression varied among the original studies. Third, the number of included studies was small, which may have contributed to the negative findings in OS and PFS. Finally, the detection method of the included studies was immunohistochemistry staining analysis, which involved the use of different antibodies and processing methods that may have provided inconsistent results for prognostic markers.

## Conclusion

In conclusion, the results of this meta-analysis showed that low *PTEN* expression was significantly associated with adverse prognosis with regard to DSS. For OS and PFS, low *PTEN* expression was not associated with unfavorable outcomes. Low *PTEN* expression presented a limited value in predicting the prognosis of patients with RCC through immunohistochemistry staining analysis. Further studies with a similar design should be conducted to confirm our results.

## Supporting information

S1 File Supporting information. Table A in S1 File. Checklist of items to include when reporting a systematic review or meta-analysis. Table B in S1 File. Characteristics of studies included in the meta-analysis. Table C in S1 File. Heterogeneity test and publication bias analyses among studies included.
(DOC)
