# Supplementary material for: Phosphatase and tensin homolog (PTEN) expression on oncologic outcome in renal cell carcinoma: A systematic review and meta-analysis
Source: PLoS One. 2017 Jul 3;12(7):e0179437. doi: 10.1371/journal.pone.0179437 (PMC5495211; doi:10.1371/journal.pone.0179437)
Supplement: S1 File — Table A in S1 File. Checklist of items to include when reporting a systematic review or meta-analysis. Table B in S1 File. Characteristics of studies included in the meta-analysis. Table C in S1 File. Heterogeneity test and publication bias analyses among studies included. (DOC) [file pone.0179437.s001.doc]

**phosphatase and tensin homolog (PTEN) expression on oncologic outcome in renal cell carcinoma: a systematic review and meta-analysis**

**supporting information**

**Table A - Checklist of items to include when reporting a systematic review or meta-analysis**

| **Section/topic** | **#** | **Checklist item** | **Reported on page #** |
| --- | --- | --- | --- |
| **TITLE** | | |  |
| Title | 1 | Identify the report as a systematic review, meta-analysis, or both. | Page 1 |
| **ABSTRACT** | | |  |
| Structured summary | 2 | Provide a structured summary including, as applicable: background; objectives; data sources; study eligibility criteria, participants, and interventions; study appraisal and synthesis methods; results; limitations; conclusions and implications of key findings; systematic review registration number. | Page 2 |
| **INTRODUCTION** | | |  |
| Rationale | 3 | Describe the rationale for the review in the context of what is already known. | Page 3,4 |
| Objectives | 4 | Provide an explicit statement of questions being addressed with reference to participants, interventions, comparisons, outcomes, and study design (PICOS). | Page 3,4 |
| **METHODS** | | |  |
| Protocol and registration | 5 | Indicate if a review protocol exists, if and where it can be accessed (e.g., Web address), and, if available, provide registration information including registration number. |  |
| Eligibility criteria | 6 | Specify study characteristics (e.g., PICOS, length of follow-up) and report characteristics (e.g., years considered, language, publication status) used as criteria for eligibility, giving rationale. | Page 4,5 |
| Information sources | 7 | Describe all information sources (e.g., databases with dates of coverage, contact with study authors to identify additional studies) in the search and date last searched. | Page 4 |
| Search | 8 | Present full electronic search strategy for at least one database, including any limits used, such that it could be repeated. | Page 4 |
| Study selection | 9 | State the process for selecting studies (i.e., screening, eligibility, included in systematic review, and, if applicable, included in the meta-analysis). | Page 4,5 |
| Data collection process | 10 | Describe method of data extraction from reports (e.g., piloted forms, independently, in duplicate) and any processes for obtaining and confirming data from investigators. | Page 5,6 |
| Data items | 11 | List and define all variables for which data were sought (e.g., PICOS, funding sources) and any assumptions and simplifications made. | Page 5,6 |
| Risk of bias in individual studies | 12 | Describe methods used for assessing risk of bias of individual studies (including specification of whether this was done at the study or outcome level), and how this information is to be used in any data synthesis. | Page 6, |
| Summary measures | 13 | State the principal summary measures (e.g., risk ratio, difference in means). | Page 5,6 |
| Synthesis of results | 14 | Describe the methods of handling data and combining results of studies, if done, including measures of consistency (e.g., I^2^) for each meta-analysis. | Page 6, |
| Risk of bias across studies | 15 | Specify any assessment of risk of bias that may affect the cumulative evidence (e.g., publication bias, selective reporting within studies). | Page 6, |
| Additional analyses | 16 | Describe methods of additional analyses (e.g., sensitivity or subgroup analyses, meta-regression), if done, indicating which were pre-specified. | Page 6,7 |
| **RESULTS** | | |  |
| Study selection | 17 | Give numbers of studies screened, assessed for eligibility, and included in the review, with reasons for exclusions at each stage, ideally with a flow diagram. | Page 6,7  Fig 1 |
| Study characteristics | 18 | For each study, present characteristics for which data were extracted (e.g., study size, PICOS, follow-up period) and provide the citations. | Page 7  Table B in S1 File |
| Risk of bias within studies | 19 | Present data on risk of bias of each study and, if available, any outcome level assessment (see item 12). | Page  8,9,10, S3 Table |
| Results of individual studies | 20 | For all outcomes considered (benefits or harms), present, for each study: (a) simple summary data for each intervention group (b) effect estimates and confidence intervals, ideally with a forest plot. | Table B and C in S1 File |
| Synthesis of results | 21 | Present results of each meta-analysis done, including confidence intervals and measures of consistency. | Page 8,9,10  Figs 2-4  Table 1and Table 2 |
| Risk of bias across studies | 22 | Present results of any assessment of risk of bias across studies (see Item 15). | Page 9,10, Fig 5 |
| Additional analysis | 23 | Give results of additional analyses, if done (e.g., sensitivity or subgroup analyses, meta-regression [see Item 16]). | Page 9,10, Fig 6 |
| **DISCUSSION** | | |  |
| Summary of evidence | 24 | Summarize the main findings including the strength of evidence for each main outcome; consider their relevance to key groups (e.g., healthcare providers, users, and policy makers). | Page 9,10 |
| Limitations | 25 | Discuss limitations at study and outcome level (e.g., risk of bias), and at review-level (e.g., incomplete retrieval of identified research, reporting bias). | Page 14 |
| Conclusions | 26 | Provide a general interpretation of the results in the context of other evidence, and implications for future research. | Page 14,15 |
| **FUNDING** | | |  |
| Funding | 27 | Describe sources of funding for the systematic review and other support (e.g., supply of data); role of funders for the systematic review. |  |

*From:*  Moher D, Liberati A, Tetzlaff J, Altman DG, The PRISMA Group (2009). Preferred Reporting Items for Systematic Reviews and Meta-Analyses: The PRISMA Statement. PLoS Med 6(7): e1000097. doi:10.1371/journal.pmed1000097

**Table B:** Characteristics of studies included in the meta-analysis

| **author** | **Publication**  **year** | **Country** | **Number of renal**  **cell carcinoma**  **patients** | **Age**  **(median)** | **Tumor size**  **(mean)** | **Follow-up**  **time (median)/**  **months** | **Survival analysis** | **Antibody used**  **for evaluation** | **Antibody**  **dilution** | **Staining**  **pattens** | **Cut-off value**  **protein positivity (%)** | **NOS** |
| --- | --- | --- | --- | --- | --- | --- | --- | --- | --- | --- | --- | --- |
| Chaoyang Zhu(1) | 2015 | China | 87 | 53.4 | NA | NA | OS | rabbit anti-human PTEN  monoclonal antibody; Boster, China | NA | cytoplasm | >1% | 9 |
| Martina Hager(2) | 2007 | Austria | 440 | 64 | NA | NA | OS | rabbit anti-PTEN antibody ab2979;  Abcam, UK | 1:500 | cytoplasm，  nuclear | >0% | 8 |
| Tobias Klatte(3) | 2008 | American | 158 | NA | NA | 38 | DSS | Zymed | 2ug/ml; |  | 100% | 8 |
| Alcides Chaux(4) | 2013 | American | 33 | 61 | 4.7 | 55 |  | D4.3 cell signaling | 1:100 | cytoplasm | H-score<10 | 8 |
| Masatomo Nishikawa(5) | 2014 | Japan | 48 | NA | NA | 13.8 | PFS | mouse monoclonal antibody;  Abcam ,UK | NA | cytoplasm | score>6 | 7 |
| Masatomo Nishikawa(6) | 2013 | Japan | 137 | 65 | NA | 63.5 | RFS | mouse monoclonal antibody;  Abcam ,UK | NA | cytoplasm | score>6 | 8 |
| JI SHIN LEE(7) | 2003 | Korea | 67 | 55 |  | 54 | OS | Neo Markers; Fremont, CA | 1:100 | cytoplasm | >0% | 7 |
| Alcides Chaux(8) | 2012 | American | 54 | 61.5 | 4.8 | 60 | OS,DSS,PFS | D4.3 cell signaling; Beverly, MA | 1:100 | cytoplasm | H-score=10 | 9 |
| Allan J. Pantuck(9) | 2007 | American | 375 | 61 | 6.5 | 56.9 | DSS | rabbit polyclonal antibody PN37 ;  Zyme San Francisco, Calif | 2 ug/mL | cytoplasm | 75%(all patients);  35%(metastatic RCC) | 8 |
| Inkeun Park(10) | 2015 | Korea | 123 | 57 | NA | 60 | PFS | Abcam, CA | NA | cytoplasm | 45% | 8 |
| Hyung L. Kim(11) | 2004 | American | 318 | 61 | 7.4 | 28 | DSS | rabbit polyclonal antibody PN37  (Zymed, South San Francisco, CA) | 2 ug/mL | cytoplasm | >50% | 9 |
| Ahmed Q. Haddad(12) | 2015 | Multicentre  American-European | 528 | 63.8 | NA | 56.5 | RFS,DSS | clone 6H2.1 Dako, Carpinteria, CA | 1：100 | cytoplasm | H-score>3 | 9 |

OS, overall survival; PFS, progression free survival; DSS, disease specific survival.

**Table C**: Heterogeneity test and publication bias analyses among studies included

|  | **Overall Survival**  **(survive vs. death)** | | | | **Progression Free Survival**  **(survive vs. death)** | | | | **Disease Specific Survival**  **(survive vs. death)** | | | | | |
| --- | --- | --- | --- | --- | --- | --- | --- | --- | --- | --- | --- | --- | --- | --- |
|  | Heterogeneity | | Publication bias | | Heterogeneity | | Publication bias | | Heterogeneity | | | Publication bias | | |
|  | P^a^ | I^2^(%)^b^ | P^c^ | P^d^ | P^a^ | I^2^(%)^b^ | P^c^ | P^d^ | | P^a^ | I^2^(%)^b^ | | P^c^ | P^d^ |
| **Over all** | 0.034 | 61.60 | 0.624 | 0.557 | 0.041 | 59.8 | 0.624 | 0.158 | | 0 | 80.1 | | 0.142 | 0.057 |
| **Geographic area** |  |  |  |  |  |  |  |  | |  |  | |  |  |
| 1. Asia | 0.132 | 55.80 | 0.317 | NA | 0.496 | 0 | 0.602 | 0.782 | | NA | NA | | NA | NA |
| 2. Non-Asian | 0.279 | 21.70 | 0.602 | 0.535 | 0.867 | 0 | 0.317 | NA | | NA | NA | | NA | NA |
| **Staining pattern** |  |  |  |  |  |  |  |  | |  |  | |  |  |
| 1. cytoplasm | 0.037 | 64.7 | 0.042 | 0.157 | NA | NA | NA | NA | | NA | NA | | NA | NA |
| 2. cytoplasm and nuclear | NA | NA | NA | NA | NA | NA | NA | NA | | NA | NA | | NA | NA |
| **Cutoff of staining** |  |  |  |  |  |  |  |  | |  |  | |  |  |
| 1. <50% | 0.025 | 73 | 0.602 | 0.8 | 0.271 | 23.3 | 0.497 | 0.795 | | NA | NA | | NA | NA |
| 2. ≥50% | 0.424 | 0 | 0.317 | NA | NA | NA | NA | NA | | 0 | 84.8 | | NA | NA |
| **Sample size** |  |  |  |  |  |  |  |  | |  |  | |  |  |
| 1. <100 | 0.037 | 64.7 | 0.042 | 0.157 | 0.42 | 0 | 0.317 | NA | | NA | NA | | NA | NA |
| 2. ≥100 | NA | NA | NA | NA | 0.142 | 48.8 | 0.117 | 0.102 | | 0.1 | 52.1 | | NA | NA |
| **Follow time(month)** |  |  |  |  |  |  |  |  | |  |  | |  |  |
| 1. <60 | 0.037 | 69.7 | 0.117 | 0.286 | 0.438 | 0 | 0.317 | NA | | 0.1 | 52.1 | | NA | NA |
| 2. ≥60 | NA | NA | NA | NA | 0.01 | 78.4 | 0.602 | 0.276 | | NA | NA | | NA | NA |
| **Patient type** |  |  |  |  |  |  |  |  | |  |  | |  |  |
| 1. localized | NA | NA | NA | NA | 0.579 | 0 | NA | NA | | NA | NA | | NA | NA |
| 2. metastatic | NA | NA | NA | NA | NA | NA | NA | NA | | NA | NA | | NA | NA |
| **Pathological type** |  |  |  |  |  |  |  |  | |  |  | |  |  |
| 1. clear cell RCC | NA | NA | NA | NA | NA | NA | NA | NA | | 0.464 | 0 | | 0.317 | NA |
| 2. other type RCC | NA | NA | NA | NA | NA | NA | NA | NA | | 0.001 | 90.8 | | 0.317 | NA |

1. Zhu C, Wei J, Tian X, Li Y, Li X. Prognostic role of PPAR-gamma and PTEN in the renal cell carcinoma. International journal of clinical and experimental pathology. 2015;8(10):12668-77.

2. Hager M, Haufe H, Kemmerling R, Mikuz G, Kolbitsch C, Moser PL. PTEN expression in renal cell carcinoma and oncocytoma and prognosis. Pathology. 2007;39(5):482-5.

3. Klatte T, Pantuck AJ, Said JW, Seligson DB, Rao NP, LaRochelle JC, et al. Cytogenetic and molecular tumor profiling for type 1 and type 2 papillary renal cell carcinoma. Clinical cancer research : an official journal of the American Association for Cancer Research. 2009;15(4):1162-9.

4. Chaux A, Albadine R, Schultz L, Hicks J, Carducci MA, Argani P, et al. Dysregulation of the mammalian target of rapamycin pathway in chromophobe renal cell carcinomas. Human pathology. 2013;44(10):2323-30.

5. Nishikawa M, Miyake H, Harada K, Fujisawa M. Expression level of phosphorylated-4E-binding protein 1 in radical nephrectomy specimens as a prognostic predictor in patients with metastatic renal cell carcinoma treated with mammalian target of rapamycin inhibitors. Medical oncology (Northwood, London, England). 2014;31(1):792.

6. Nishikawa M, Miyake H, Harada K, Fujisawa M. Expression of molecular markers associated with the mammalian target of rapamycin pathway in nonmetastatic renal cell carcinoma: Effect on prognostic outcomes following radical nephrectomy. Urologic oncology. 2014;32(1):49.e15-21.

7. Shin Lee J, Seok Kim H, Bok Kim Y, Cheol Lee M, Soo Park C. Expression of PTEN in renal cell carcinoma and its relation to tumor behavior and growth. Journal of surgical oncology. 2003;84(3):166-72.

8. Chaux A, Schultz L, Albadine R, Hicks J, Kim JJ, Allaf ME, et al. Immunoexpression status and prognostic value of mammalian target of rapamycin and hypoxia-induced pathway members in papillary cell renal cell carcinomas. Human pathology. 2012;43(12):2129-37.

9. Pantuck AJ, Seligson DB, Klatte T, Yu H, Leppert JT, Moore L, et al. Prognostic relevance of the mTOR pathway in renal cell carcinoma: implications for molecular patient selection for targeted therapy. Cancer. 2007;109(11):2257-67.

10. Park I, Cho YM, Lee JL, Ahn JH, Lee DH. Prognostic tissue biomarker exploration for patients with metastatic renal cell carcinoma receiving vascular endothelial growth factor receptor tyrosine kinase inhibitors. Tumour biology : the journal of the International Society for Oncodevelopmental Biology and Medicine. 2016;37(4):4919-27.

11. Kim HL, Seligson D, Liu X, Janzen N, Bui MH, Yu H, et al. Using protein expressions to predict survival in clear cell renal carcinoma. Clinical cancer research : an official journal of the American Association for Cancer Research. 2004;10(16):5464-71.

12. Haddad AQ, Kapur P, Singla N, Raman JD, Then MT, Nuhn P, et al. Validation of mammalian target of rapamycin biomarker panel in patients with clear cell renal cell carcinoma. Cancer. 2015;121(1):43-50.
